# Evaluation of the Persian version of modified fatigue impact scale in Iranian patients with multiple sclerosis

**Published:** 2013

**Authors:** Mohammad Hossein Harirchian, Somayeh Nasergivechi, Marzieh Maddah, Alipasha Meysamie, Homayoun Amini, Ehsan Esmaelii Shandiz, Abbas Tafakhori

**Affiliations:** 1 Professor, Iranian Center of Neurological Research, Department of Neurology, School of Medicine, Tehran University of Medical Sciences, Tehran, Iran; 2 Immunology, Allergy and Asthma Research Institute, Tehran University of Medical Sciences, Tehran, Iran; 3 Associate Professor, Department of Community and Preventive Medicine, School of Medicine, Tehran University of Medical Sciences, Tehran, Iran; 4 Associate Professor, Psychiatry and Psychology Research Center, Tehran University of Medical Sciences, Tehran, Iran; 5 Iranian Center of Neurological Research, Department of Neurology, School of Medicine, Tehran University of Medical Sciences, Tehran, Iran; 6 Assistant Professor, Iranian Center of Neurological Research, Department of Neurology, School of Medicine, Tehran University of Medical Sciences, Tehran, Iran

**Keywords:** Multiple Sclerosis, Fatigue, Persian Translation, Modified Fatigue Impact Scale

## Abstract

**Background:**

Fatigue, a major cause of disability in individuals with multiple sclerosis (MS), is associated with reduced quality of life. The aim of this study was to evaluate the reliability and reproducibility of the Persian version of Modified Fatigue Impact Scale (MFIS) in Iranian patients with MS.

**Methods:**

This study included 15 subjects with clinically definite MS, 15 hospitalized patients with MS, and 15 hospitalized patients with other chronic illnesses (as controls). They filled in the Persian version of the MFIS twice with a three-day interval. MFIS items were analyzed and the correlation coefficient was calculated.

**Results:**

There was a good correlation between the scores of the two measurements (correlation coefficient: 0.984, P < 0.001) especially in physical and cognitive subgroups. The reproducibility of psychosocial subscale was lower than physical and cognitive subscales.

**Conclusion:**

According to our findings, the Persian version of the MFIS has a good reliability and reproducibility for assessment of fatigue in patients with MS.

## Introduction

Chronic fatigue is a common symptom in multiple sclerosis (MS) patients, which interferes with, and considerably limits their daily activities. 15% to 40% of MS patients report it as the most disabling MS symptom and more than 65% usually experience fatigue during afternoons.^1^

Fatigue can be influenced by motor disabilities, painful syndromes, and mood swings,^2^ but it might happen at any stage of the disease, independent of those factors. This symptom is more frequent and severe in primary and secondary progressive patients than in relapsing remitting patients.^3^

MS fatigue is difficult to define. Its etiology and pathophysiology are known to a very small degree. Both peripheral and central mechanisms may have a role, but there are no biologic or neuroimaging markers known for it up to this date. That is why it is one of the most challenging MS symptoms to treat.^4^

MS Patients feel that the effort needed to do an activity is disproportionately high.^5^ Therefore, they would rather minimize their physical activities, but even when resting it does not help them much.^5^ The symptom of fatigue affects individuals’ cognitive functions too; decreasing their concentration and attention. Therefore, fatigue is one of the main reasons for MS individuals to quit their jobs. All in all, fatigue can make MS patients’ daily and social lives harder and limit their working abilities.^6^

Studies have shown that psychological and physical factors can worsen the clinical picture of fatigue in MS patients. Examples of which include depression, stress, sleeplessness, heat intolerance, decreased mobility, work-related problems, and infections.^7^

The Modified Fatigue Impact Scale (MFIS), suggested by the Multiple Sclerosis Council for clinical practice guidelines, has been a reliable tool for assessing MS fatigue. MFIS is derived from the 40-item Fatigue Impact Scale (FIS), and contains only 21 items, but offers a more multidimensional assessment: physical (pMFIS, 9 items), psychosocial (psMFIS, 2 items), and cognitive (cMFIS, 10 items) functioning. The sum of the 21 items makes the total score. MFIS is easy to use, and has other advantages such as good reproducibility and strong correlation with FIS results (r = 0.68).^8^

This scale has not been previously validated in Persian language; in this study, we evaluated the Persian translation of MFIS in Iranian MS patients.

## Materials and Methods

The approval of this study was obtained from Tehran University of Medical Sciences and by the Ethnic Committee.

At first, the MFIS was translated to Persian by two different translators and then the Persian guidelines retranslated to English for comparison in which there was no significant difference between them.

15 subjects with clinically definite MS by McDonald Criteria, 15 hospitalized MS patients, and 15 hospitalized patients with other chronic illnesses such as diabetes mellitus, and rheumatoid arthritis as controls were enrolled in this study. The sample of outpatients with MS was recruited primarily from an outpatient MS subspecialty clinic at Imam Khomeini hospital in Tehran, Iran.

Subjects with any MS disease subtype (relapsing-remitting MS, secondary progressive MS, and primary progressive MS) and any Expanded Disability Status Scale (EDSS) score were eligible for entry. The exclusion criteria were use of stimulants in the prior 6 weeks (modafinil, pemoline, and methylphenidate) and inability to complete the questionnaires or study outcome measures (severe mental or cognitive disability, illiteracy or severe visual impairment). The groups were matched in age and sex.

The subjects filled MFIS twice with three days interval. MFIS items were analyzed and correlation coefficient was measured. Paired sample t-test was used to compare MFIS item's means of two questioners and correlation coefficient for reproducibility of test.

## Results

Fifteen MS outpatients (mean age 32.2 ± 8.1 years), fifteen hospitalized MS patients (mean age 29.4 ± 11.2 years) and 15 hospitalized patients without MS (mean age 29.7 ± 8.9 years) were included. Physical, cognitive and psychosocial subscales of MFIS were measured and compared between groups. Table 1 summarizes the mean and standard deviation of MFIS in three groups and Table 2 shows the reproducibility of the test according to Correlation Coefficient. In Table 3 the mean of three subscales of MFIS is compared between groups.

**Table 1:** Mean and standard deviation of Modified Fatigue Impact Scale (MFIS) in three groups

Different variables	Mean ± SD		

	Hospitalized	Out-patient	Without MS
Age (years)	29.3 ± 8.1	35.2 ± 11.3	29.4 ± 8.3
MFIS – Time 1	37.1 ± 11.6	27.6 ± 18.1	21.3 ± 8.5
MFIS - Time 2	40.7 ± 13.5	28.9 ± 16.6	23.1 ± 9.8
Physical subscale - Time 1	18.1 ± 9.8	16.4 ± 10.0	9.33 ± 3.7
Physical subscale - Time 2	20.3 ± 9.9	18.3 ± 10.4	10.5 ± 4.5
Cognitive subscale - Time 1	13.8 ± 5.1	8.13 ± 9.3	8.60 ± 5.3
Cognitive subscale - Time 2	15.4 ± 6.8	6.73 ± 6.2	9.93 ± 7.2
Psychosocial subscale - Time 1	5.13 ± 2.3	3.07 ± 2.7	2.67 ± 2.0
Psychosocial sub scale - Time 2	5.00 ± 2.0	5.00 ± 5.4	2.60 ± 1.5

**Table 2:** Reproducibility of Modified Fatigue Impact Scale subscales

Modified Fatigue Impact Scale and subscales	Correlation coefficient					

	Hospitalized		Out-patient		Without MS	

	CC*	P	CC	P	CC	P
Modified Fatigue Impact Scales	0.984	< 0.001	0.842	< 0.001	0.964	< 0.001
Physical Subscale	0.878	< 0.001	0.952	< 0.001	0.901	< 0.001
Cognitive Subscale	0.856	< 0.001	0.852	< 0.001	0.846	< 0.001
Psychosocial Subscale	0.148	0.598	0.664	0.007	0.837	< 0.001

* Correlation coefficient

**Table 3:** Mean difference of two Modified Fatigue Impact Scales measurement in three groups

Modified Fatigue Impact Scale and Subscales	Mean difference between two measurements (P)		

	Hospitalized	Out-patient	Without MS
Modified Fatigue Impact Scales	-3.67 (0.25)	-1.80 (0.15)	-1.33 (0.07)
Physical subscale	-2.13 (0.45)	-1.13 (0.16)	-1.93 (0.01)
Cognitive subscale	-1.60 (0.21)	-1.33 (0.31)	1.40 (0.10)
Psychosocial subscale	0.13 (0.81)	0.07 (0.20)	-1.93 (0.77)

## Discussion

MS fatigue is still poorly understood and often underestimated for some reasons. First of all, fatigue is a subjective symptom which does not have a unified definition. Lack of energy, a feeling of exhaustion and tiredness are included in most definitions, but they may be interpreted differently depending on cultural or educational backgrounds.^9^ Another problem is that there is no gold standard to measure fatigue. Although there are questionnaires which can be helpful to rate the level of fatigue, but most of them do not include questions to qualify or define the fatigue. Furthermore, most clinicians do not know which are best suited to MS patients.^10^

As it is a sensitive area for most people to answer, reproducibility of psychosocial subscale is less than physical and cognitive subscales in this questionnaire. Furthermore there is a wide range of psychological disorders in MS which can fluctuate over time and this can lower the reproducibility of the test.

It is very important to standardize the fatigue questionnaire in Persian patients, because it is an essential part in planning further studies in evaluating fatigue and performing interventional studies.

As there are different languages and accents in Iran (such as Azeri, Kurdish, etc.) therefore such studies can be performed to evaluate reliability and reproducibility in these subgroups.

The primary and secondary fatigue conditions (due to sleep disturbance, depression, etc.) in MS patients were not discussed in this paper and further studies are necessary to evaluate this paradigm.

According to this study, Persian translation of MFIS questionnaire has a good reliability and reproducibility for assessment of fatigue in MS patients.

This study can help for better evaluation of fatigue in Persian MS patients, and therefore a better evaluation of drug effect and conducting trials in fatigue of MS patients.

## Conclusion

According to this study, Persian translation of MFIS questionnaire has a good reliability and reproducibility for assessment of fatigue in MS patients.
